# Gut Microbiota and Parasite Dynamics in an Amazonian Community Undergoing Urbanization in Colombia

**DOI:** 10.1101/2025.04.16.25325921

**Published:** 2025-04-17

**Authors:** Sebastián Díaz, Amie J. Eisfeld, Mónica Palma-Cuero, Nathalie Dinguirard, Leah A. Owens, Karl A. Ciuoderis, Laura S. Pérez-Restrepo, John D. Chan, Tony L. Goldberg, Jessica L. Hite, Juan Pablo Hernandez-Ortiz, Yoshihiro Kawaoka, Mostafa Zamanian, Jorge E. Osorio

**Affiliations:** 1 UW-GHI One Health Colombia, Universidad Nacional de Colombia, Medellín, Colombia; 2 Department of Pathobiological Sciences, University of Wisconsin-Madison, Madison, WI, USA; 3 Grupo de Estudios en Salud Pública de la Amazonía, Laboratorio de Salud Pública Departamental del Amazonas, Leticia, Colombia; 4 Global Health Institute, University of Wisconsin-Madison, Madison, WI, USA; 5 Corporacion Corpotropica, Villavicencio, Colombia; 6 Faculty of Life Sciences, Universidad Nacional de Colombia, Medellín, Colombia

## Abstract

Studies on human gut microbiota have recently highlighted a significant decline in bacterial diversity associated with urbanization, driven by shifts toward processed diets, increased antibiotic usage, and improved sanitation practices. This phenomenon has been largely overlooked in the Colombian Amazon, despite rapid urbanization in the region. In this study, we investigate the composition of gut bacterial microbiota and intestinal protozoa and soil-transmitted helminths (STHs) in both urban and rural areas of Leticia, which is located in the southern Colombian Amazon. Despite their geographic proximity, the urban population is predominantly non-indigenous, while indigenous communities mostly inhabit the rural area, resulting in notable lifestyle differences between the two settings. Our analyses reveal a reduction in bacterial families linked to non-processed diets, such as *Lachnospiraceae, Spirochaetaceae*, and *Succinivibrionaceae*, in the urban environment compared to their rural counterparts. Interestingly, *Prevotellaceae*, typically associated with non-processed food consumption, shows a significantly higher abundance in urban Leticia. STH infections were primarily detected in rural Leticia, while intestinal protozoa were ubiquitous in both rural and urban areas. Both types of parasites were associated with higher gut bacterial richness and diversity. Additionally, microbial metabolic prediction analysis indicated differences in pathways related to unsaturated fatty acid production and aerobic respiration between rural and urban bacterial microbiomes. This finding suggests a tendency towards dysbiosis in the urban microbiota, possibly increasing susceptibility to non-communicable chronic diseases. These findings provide new insights into the impact of urbanization on gut microbiota dynamics in the Amazonian context and underscore the need for further research to elucidate any associated health outcomes.

## Introduction

The taxonomic composition of the human gut microbiota is dynamic and shaped by factors such as diet, medication, sanitation, and occupational exposures [1]. Surveys on microbiota composition during population rural-to-urban transition have consistently observed a reduction in microbial diversity, particularly of bacterial groups associated with traditional lifestyles. These are known as VANISH (volatile and/or negatively associated with industrialized human societies) taxa. Examples include *Prevotellaceae, Spirochaetaceae*, and *Succinivibrionaceae* [2–5], whose decline is attributed to dietary shifts towards processed foods, widespread antibiotic usage, and decreased physical and outdoor activity. This is accompanied by a corresponding expansion of BloSSUM (bloom or selected in societies of urbanization/modernization) taxa, such as *Bacteroidaceae* and *Verrucomicrobia*, which are linked to an increased incidence of chronic disease [6,7]. These trends are expected to increase with the global rise of urbanization, with over half of the world’s population currently residing in urban areas [8].

Gut bacteria also interact with commensal and parasitic eukaryotes in the human host, including protists and soil-transmitted helminths (STHs) [9,10]. These single-celled and multicellular organisms interact with the bacterial community and are associated with increased richness and diversity, or changes in the abundance of specific bacterial taxa [11–17]. For example, abundance of the *Prevotella* genus of gut bacteria is positively associated with *Blastocystis spp*. and *Endolimax nana* protozoa [12], but negatively associated with the presence of *Entamoeba* [18]. These interactions can also be clinically relevant, such as the negative correlation between the *Megasphaera* genus of bacteria and diarrheal symptoms during *Cryptosporidium* infection [19]. Helminth parasites also alter host-microbiota interactions [20], with several well-known immunomodulatory effects of helminth infection requiring host microbiota [21,22]. Infection with *Trichuris muris* and *Heligmosomoides polygyrus* roundworms inhibits proinflammatory bacterial taxa while promoting colonization with protective *Clostridiales* species, and this relationship can be reversed with deworming [23]. The relative abundance of *Clostridiales* also changes in individuals infected with hookworm (*Necator americanus*) following anthelmintic treatment [24]. Both protozoa and helminth parasites also stimulate intestinal tuft cells [25,26]. These cells promote type II immunity and alter intestinal microbiota composition [27,28] while also secreting signals that directly modulate parasite biology [29].

Demographic and cultural change in the Amazonian region is characterized by an urban expansion of existing riverside towns and the establishment of new peri-urban settlements driven by agriculture, extractive industries, and infrastructure development [30]. However, gut microbiota studies have predominantly focused on rural horticulturists and hunter-gatherers, with limited attention given to urban populations [31–36]. Leticia, situated in the southern Colombian Amazon, serves as the capital city of the Departamento del Amazonas and forms an urban complex straddling the borders of Brazil, Colombia, and Peru. The city’s population is estimated at 100,000 inhabitants, with distinct demographic characteristics observed between urban and peri-urban rural areas [37,38]. Mixed-race populations predominantly inhabit the urban area, while indigenous groups primarily populate rural communities settled in response to extractive booms (e.g. rubber and coca) and assimilation efforts such as missionary campaigns [39].

This study provides novel insights into the urban and rural gut microbiota and parasite communities of a Colombian Amazonian population. We aim to understand how social factors, medical history, and current infection with parasitic protozoa and STHs interact with the bacterial microbiota composition. Additionally, using metabolic pathways predictions, we explore the potential health implications of the bacterial taxonomic differences between urban and rural areas.

## Materials and methods

### Ethics and Human Subjects

All work conducted in this study received approval from the Research Ethics Committee of Universidad Nacional de Colombia (protocol number CEMED-060–19), the University of Wisconsin-Madison Health Sciences Institutional Review Board (protocol number 2020–0214), and local indigenous community leaders. Healthy volunteers were recruited in March 2021 from two field sites: (a) the peri-urban rural multi-ethnic indigenous community of Nimaira Naimeki Ibiri Kilómetro 11 (referred to as Km11) (n = 80); and (b) within the urban city limits of Leticia (referred to as Leticia) (n = 20). Colombian Army and Air Force support facilitated operations at the Km11 field site. Before enrollment, consent was obtained from all participants. For individuals under 18 years old, formal written consent was provided by their parents or legal guardians. Before sampling, each participant completed a survey addressing socioeconomic and health status.

### Donor sampling

Enrolled participants were provided with a specialized kit for fecal specimen collection, along with verbal instructions for proper sample acquisition. Upon collection, fecal specimens were promptly stored in pack ice for transportation to the State Public Health Laboratory. Aliquots of 1 g of the specimens were preserved in 2 ml of DNA/RNA Shield (Zymo Research) and stored at −80°C. Preserved specimens were transferred to the One Health Genomic Laboratory (OHGL) at the Universidad Nacional Sede Medellín and shipped to the University of Wisconsin-Madison for DNA extraction and sequencing. In addition to fecal specimens, serum samples and nasal swabs were collected for SARS-CoV-2 testing. Recent infections were assessed using the Abbott Architect SARS-CoV-2 IgG antibody assay in serum (Abbott Park, IL). Active infections were determined through genomic DNA extraction from nasal swabs using Gene E RT-PCR, as previously described [40].

### DNA Extraction

DNA extraction from fecal specimens was conducted using the QIAamp PowerFecal Pro DNA kit (Qiagen). The extraction process followed the manufacturer’s protocol, with a modification at Step 1, where the input material consisted of 50 μl of fecal slurry in DNA/RNA Shield, 500 μl of CD1 buffer, and 300 μl of ATL buffer (Qiagen; not included in the kit). Bead beating was performed using a TissueLyser II (Qiagen) for two cycles, each lasting 5 minutes at 25 Hz. Between cycles, adaptors containing the specimens were repositioned so that samples that were closer to the machine body were further away in the second cycle. Finally, samples were eluted in a final volume of 50 μl RNase-free water and stored at −80°C.

### 16s rRNA metabarcoding sequencing and analysis

Qiagen Genomic Services conducted 16S rRNA microbiome profiling using the QIAseq 16S/ITS Screening Panel for library preparation. First, starting with 1 ng of DNA, target regions were selected and amplified through 12 cycles of PCR. Samples underwent cleanup using QIAseq Beads (Qiagen), followed by the addition of sequencing adapters and enrichment in a second PCR of 12 cycles. After a second bead cleanup, the libraries underwent quality control assessment using capillary electrophoresis (Tape D1000). High-quality libraries were then pooled in equimolar concentrations, determined by the Bioanalyzer automated electrophoresis system (Agilent Technologies). The library pool(s) were quantified using qPCR, and the optimal concentration was used to generate clusters on the surface of a flow cell before sequencing on a MiSeq (Illumina Inc.) instrument (2×276). Sequencing data were deposited in the NCBI SRA database (bioproject accession number PRJNA1246579).

Raw V4-V5 16S rRNA fragment reads were processed using a QIIME2 pipeline [41]. The DADA2 plugin [42] was utilized for trimming reads, removing sequences with ambiguous nucleotides and chimeras, and discarding singletons. The remaining sequences, with a length of approximately 370 bp, were clustered into Operational Taxonomic Units (OTUs) at a 99% identity level. Taxonomic classification was performed using the q2-feature-classifier plugin with the Bayes machine-learning classifier method [43] trained with the Greengenes 515F/806R database v.13.8 [44] with OTUs identified as Mitochondria, Chloroplast, or Archaea discarded. After removing low abundance OTUs (≤ 0.01% total sampling) and samples (≤ 1000 sequences), two individuals from Km11 were discarded, resulting in a final sample size of 98 individuals.

Alpha and beta diversity analyses were conducted in R in the phyloseq package [45]. To assess the overall influence of donor location, a permutational multivariate analysis of variance (PERMANOVA) with 999 permutations was performed on weighted UniFrac distances using the vegan package [46]. To identify microbial composition differences between the locations, two approaches were employed: (a) identify OTUs with differential abundance using the Wald significance test implemented in DESeq2 [47], with Km11 as the treatment group and Leticia as the control (threshold cutoff, α = 0.01); (b) evaluate the differential abundance of selected BloSSUM and VANISH taxa by using Wilcoxon paired tests. For BloSSUM taxa, *Bacteroidaceae*, *Verrucomicrobiaceae*, and *Rikenellaceae*, were chosen. For for the VANISH taxa, *Prevotellaceae* plus *Paraprevotellaceae* (referred as *Prevotellaceae*), *Succinivibrionaceae*, *Spirochaetaceae*, and the clostridiales *Lachnospiraceae* with *Ruminococcaceae* as one group (referred as *Lachnospiraceae*) were chosen. For microbiota functional analysis, PICRUSt 2.0 [48] was utilized to predict biological pathways. Results were classified with the MetaCyc database [49] and differential abundances between Km11 and Leticia microbial pathways were evaluated using a Wald significance test within the ggpicrust2 package [50].

To describe the Leticia bacterial microbiota structure in a regional context, we performed a comparative analysis of our dataset against previously reported Amazonian and non-Amazonian Colombian microbiotas. We included datasets that meet two criteria: used the V4 region of the 16S rRNA sequence and have reads with >100 bp in length. The final analysis included nine datasets, divided into six Amazonian populations: (a) the urban and rural Leticia sampling as one group; (b) the urban Belém and rural indigenous (c) Suruí, (d) Tupaiú, and (e) Xikrin [4] communities in Brazil; and the (f) rural indigenous Tsimané community in Bolivia [34]; and three non-Amazonian datasets, two urban Colombian (g) Bogotá and (h) Medellín) [45]; and (i) an urban American cohort from Ohio, subsampled from the American Gut Project [46] (**Table S1**). Given that some datasets only sampled adult donors, infant samples (<15 years old) were removed before the analysis. Raw 16S rRNA reads were trimmed to 130 bp and preprocessed using the QIIME2 pipeline. Comparative analysis of alpha and beta diversity and selected BloSSUM and VANISH taxon abundance were performed in R.

### 18s rRNA metabarcoding sequencing and analysis

For the same DNA samples used for 16s rRNA analysis, we carried out eukaryotic analysis using the VESPA (Vertebrate Eukaryotic Endo-Symbiont and Parasite Analysis) metabarcoding protocol [51] targeting the 18S rRNA gene V4 region. Library pools were sequenced using MiSeq (Illumina Inc.) instrument with a 300×300 cycle chemistry. Sequencing data were deposited in the NCBI SRA database (bioproject accession number PRJNA1246579). Raw reads were processed using a QIIME2 pipeline with OTUs at a 99% identity level classified using the PR2 reference sequence database v.5.0 [52]. OTUs with unassigned or incomplete taxonomy using the PR2 database were manually classified using the full NCBI nucleotide database. Three Km11 samples with ≤ 1000 sequences were discarded from the final dataset. PERMANOVA tests were used to evaluate the influence of parasitic protists and nematodes in bacterial community structure.

## Results

We evaluate the impact of urbanization on local microbiota structure in the gut microbiota of Leticia by comparing the peri-urban rural indigenous community of Kilometro 11 (Km11) with the non-indigenous urban population (Leticia). Participants completed surveys that provided insights into the conditions of the community (Table 1). The cohort showed a representative sex distribution (Leticia, 60% female, 40% male; Km11, 58% female, 42% male) with donors ranging from 5 to 82 years (Leticia, median age 36.5 years old; Km11 38.0 years old). As expected, ethnic identification varied significantly by location, with 95% of the Km11 community identifying as indigenous (primarily Wuitoto, Ticuna, and Murui). In contrast, only one donor from Leticia identified as indigenous, with most self-describing as *mestizos* (mixed-race). Most Km11 participants typically spend the day around their residence with high contact with livestock (82%), raising and sacrificing mostly poultry (chickens and ducks). Conversely, companion animal contact showed no significant difference between sites. Finally, over 90% of the donors stored water at home for domestic use.

Both groups reported low frequencies of historical diagnoses of vector-borne and chronic diseases (18–35%, with cardiovascular disease and hypertension being the most prevalent). Over 50% of donors at both locations had recent SARS-CoV-2 infection indicated by an IgG antibody assay, and less than 10% had an active positive infection based on RT-PCR (Table 1) although they were asymptomatic at the time of sampling. Medication use reported during the preceding month was high, particularly in Km11, especially for analgesics like paracetamol and ibuprofen.

**Fig 1.**
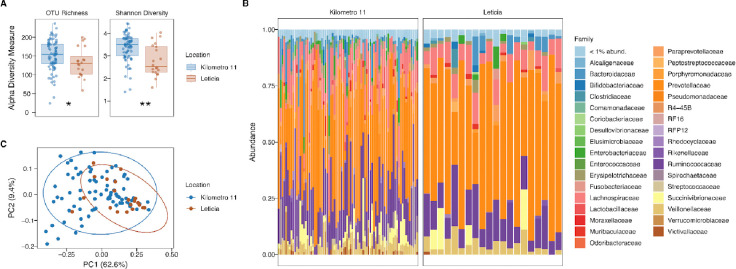
Leticia gut bacteria microbiota alpha and beta diversity analysis. **(A)** Alpha diversity estimators, (a) Number of OTUs (richness) and (b) Shannon Index (diversity). Difference between locations evaluated using Wilcoxon Paired-tests. *p = 0.05–0.005, **p = 0.0049–0.0005, ***p < 0.00049. **(B)** Relative bacterial family abundance for locations. Families with < 1% abundance were merged into one group. **(C)** PCA plot of bacterial community structure based on the weighted UniFrac distances.

To describe the bacterial microbiota compositions of the two groups, we sequenced the V4-V5 16S rRNA region. After preprocessing the reads, our final metabarcoding dataset comprised an average of 28,798 ± 15,564 sequences per sample, distributed across 466 OTUs. Most samples exhibited good taxonomic coverage, reaching the maximum number of OTUs with a subsampling of 10,000 sequences (Fig. S1). Both richness (number of OTUs, p value = 0.0449) and diversity (Shannon index, p value = 0.001179) were statistically significantly reduced in the Leticia group (Fig. 1A). At the family level, *Prevotellaceae* was the most abundant group in the sampling, with >38% of the overall abundance for both locations (Fig. 1B), followed by *Ruminococcaceae* (~15%). We evaluated the influence of the surveyed variables on microbiota structure using a PERMANOVA analysis. The results revealed a statistically significant influence of location (F = 7.904, R^2^ = 0.08; p = 0.002), with the Leticia samples clustering in the PCA plot (Fig. 1C). Other variables also differed between locations, such as ethnic identification, livestock contact, mosquito net use, healthcare coverage, and educational level, and these were significantly associated with the gut microbiota community structure (Table 1).

**Fig 2.**
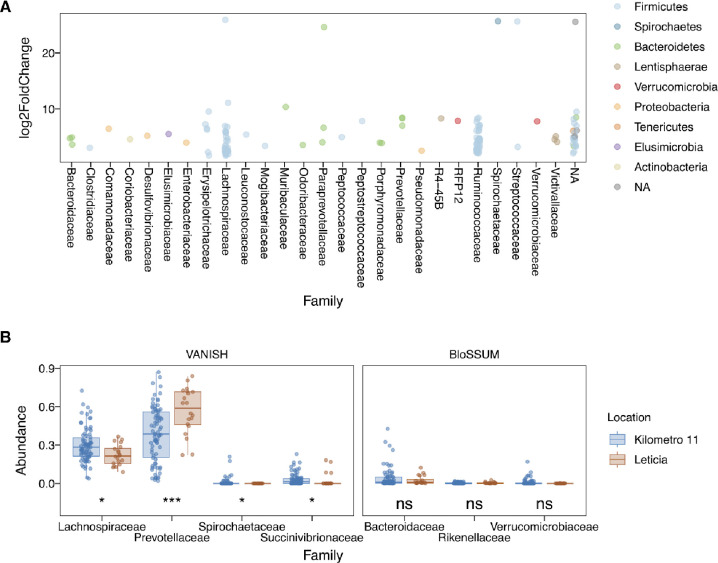
Differential abundance analysis between Leticia and Km11. **(A)** Significant OTUs according to the Wald significance test. Km11 as the treatment and Leticia as the control group, with an α = 0.01 threshold cutoff. **(B)** Differential abundance of VANISH and BloSSUM taxa. Difference between locations evaluated using Wilcoxon paired-tests. *p = 0.05–0.005, **p = 0.0049–0.0005, ***p < 0.00049.

Next, to identify the taxa driving differences between Leticia and Km11, we analyzed bacterial differential abundance using two approaches. For the Wald significance test, 135 out of 466 total OTUs showed a statistically significant log fold change (**Table S2**), with increased abundance in Km11 compared to Leticia (Fig. 2A). Most of these OTUs (55%) belong to the clostridial families *Ruminococcaceae* and *Lachnospiraceae*, and 22% were absent in Leticia samples (**Table S2**). The selected family abundance analysis (Fig. 2B) showed significant reductions of VANISH taxa in Leticia, with *Spirochaetaceae* locally extinct and only *Prevotellaceae* increased in the urban setting. No significant difference was observed for BloSSUM groups, being in low abundance for most donors.

**Fig 3.**
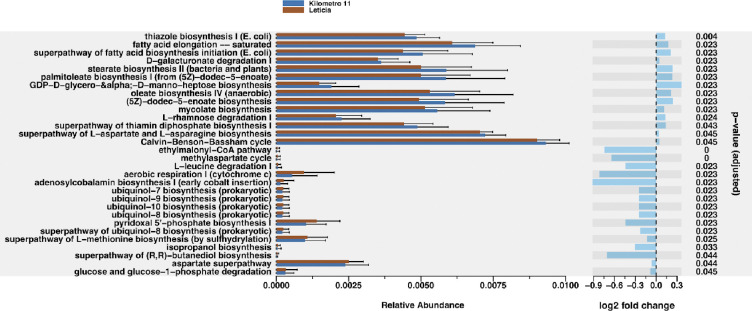
Predictive bacterial metabolic pathways analysis. **(Left)** Relative abundance of pathways with differential abundance between locations. **(Right)** Logarithmic fold change between pathways based on Wald Significance Test. Km11 as the treatment and Leticia as the control group, with an α = 0.01 threshold cutoff.

In order to infer physiological implications of these bacterial microbial repertoires, we performed a predictive functional analysis finding a total of 331 predicted metabolic pathways. Using a Wald significance test, we identified 30 pathways with significant differences in relative abundance between the two locations (Fig. 3 and **Table S3**). Compared to Leticia, 14 pathways were increased in Km11, with half of these belonging to fatty acid and lipid biosynthesis pathways. A total of 16 pathways were increased in Leticia, including pathways associated with aerobic respiration like cofactors biosynthesis pathways.

**Fig 4.**
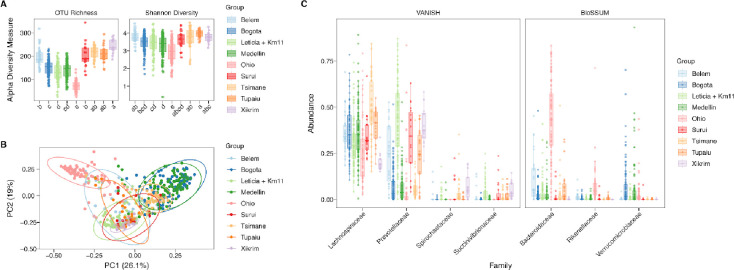
Amazonian and non-Amazonian gut bacteria microbiota datasets alpha and beta diversity comparative analysis. **(A)** Alpha diversity estimators of number of OTUs (richness) and Shannon Index (diversity). Letters reflect grouping and differences between datasets evaluated using Tukey HSD test. **(B)** PCA plot of bacterial community structure based on the weighted UniFrac distances. **(C)** Abundance of VANISH and BloSSUM taxa across bacteria microbiota datasets.

To evaluate the bacterial community compositions in a regional context, we performed a comparative gut bacteria microbiota analysis comprising nine datasets, including six Amazonian datasets (two urban groups including our sampling, and four rural groups) and three urban non-Amazonian datasets, for a total of 489 samples (**Table S1**; Fig. S2). For the alpha diversity (Fig. 4A), rural Amazonian groups showed higher richness than urban communities, where Leticia has similar values to the other Colombian urban microbiotas. We performed a PERMANOVA analysis to evaluate how much the sampled location can explain the microbiota structure (F = 37.94, R^2^ = 38.09; p = 0.001). This was visualized in the PCA, where Ohio samples formed a separated cluster from three South American subgroups, Colombian urban Medellín-Bogotá, one transitional group for the Amazonian rural Tsimané, and the Leticia samples indistinct from Brazilian rural and urban populations (Fig. 4B). The clustering of Leticia and Brazilian samples is explained in the selected family analysis (Fig. 4C), where VANISH taxa *Prevotellaceae* and *Succinivibrionaceae* are increased compared to the other populations, while BloSSUM groups dominated in the urban populations (*Bacteroidaceae* and *Rikenellaceae* in Ohio, and *Verrucomicrobiacea* in Bogotá and Medellín) are reduced. Belém, the other Amazonian city evaluated, is distinguished from Leticia for the higher abundance of *Bacteroidaceae,* even with some samples clustering with the Ohio samples.

**Fig 5.**
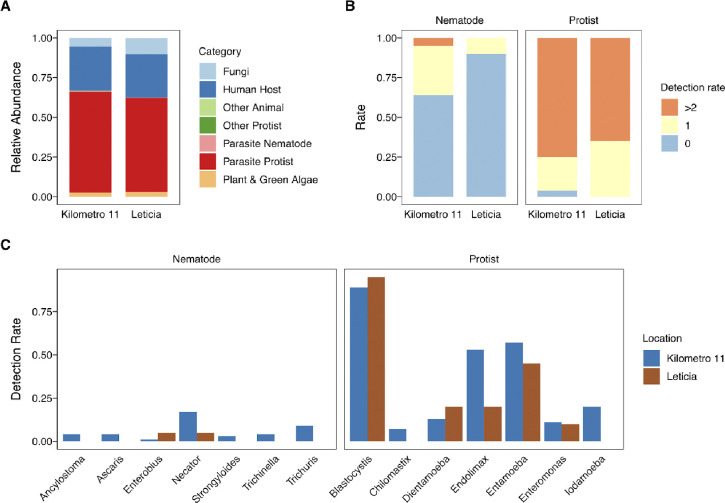
Leticia eukaryotic parasite metabarcoding description. **(A)** Relative sequence abundance from the 18s rRNA metabarcoding for each taxonomic category. **(B)** Overall detection rate of parasite nematodes and protists in Km11 and Leticia samples and **(C)** breakdown of taxa detected.

To describe the parasitic protists and nematodes associated with the Leticia gut microbiota, we performed an 18s rRNA analysis using the VESPA (Vertebrate Eukaryotic endoSymbiont and Parasite Analysis) protocol for eukaryotic endosymbiont metabarcoding [51]. Amplicons were generated targeting the 18S rRNA gene V4 region, sequenced, and recovered reads were classified into seven taxonomic categories using the PR2 reference sequence database. Parasitic protist sequences were the most common group, followed by human host. Parasitic nematodes were recovered in a low abundance (<1%) (Fig. 5A). Most individuals in both locations were infected with two or more parasitic protist taxa (Fig. 5B). Nematode infections were mostly detected in rural Km11 (Fig. 5B). Only two nematode taxa, *Enterobius* and *Necator*, were detected in urban Leticia, while seven genera were found in Km11 at rates ranging from 1 to 17% (Fig. 5C). Five protist taxa were detected in both locations, with *Blastocytis* being the most common, detected in >85% of samples from both locations (Fig. 5C).

**Fig 6.**
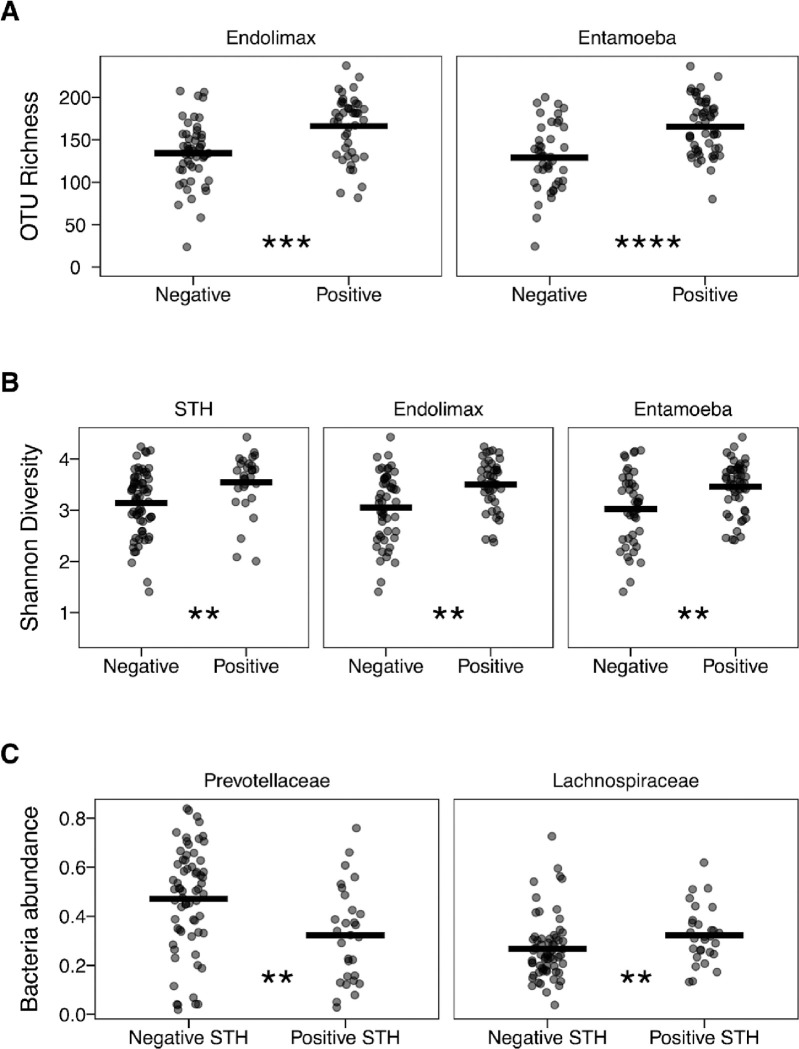
Differential analysis between parasite positive and negative Leticia (both urban and rural) samples. **(A)** Richness (observed number of OTUs) for *Endolimax* and *Entamoeba* infection. **(B)** Diversity (Shannon index) for STH, *Endolimax*, and *Entamoeba* infection. **(C)** *Prevotellaceae* and *Lachnospiraceae* abundance for STH infection. Difference between infection status evaluated using Wilcoxon paired-tests. *p = 0.05–0.005, **p = 0.0049–0.0005, ***p < 0.00049.

Given the extensive literature indicating that parasite infection influences the host microbial environment, we tested whether there was an association with protist or helminth infection and either bacterial diversity and abundance. PERMANOVA tests were used to evaluate alpha diversity metrics for the number of observed OTUs (richness), Shannon index (diversity), and abundance of *Prevotellaceae* and *Lachnospiraceae* family of bacteria. The Leticia and Km11 datasets were combined into one group, and comparisons were made between positive and negative infection status with any STH taxa or infection with any of the five most prevalent protists (*Blastocystis*, *Dientamoeba*, *Endolimax*, *Entamoeba*, and *Enteromonas*). Parasite infection was significantly associated with increased richness of bacterial tax for *Endolimax* (F = 12.137, R^2^ = 0.115, p = 0.001) and *Entamoeba* (F = 19.834, R^2^ = 0.176, p = 0.001). Infection with the following protist and nematode parasites were associated with increased diversity; *Endolimax* (F = 12.618, R^2^ = 0.119, p = 0.001), *Entamoeba* (F = 12.625, R^2^ = 0.119, p = 0.003), and STH infection (F = 6.919, R^2^ = 0.069, p = 0.007). For the bacterial taxonomic abundance, STH infection influenced both *Prevotellaceae* (F = 6.722, R^2^ = 0.068, p = 0.001) and *Lachnospiraceae* (F = 4.753, R^2^ = 0.048, p = 0.018). All the results were confirmed with Wilcoxon paired tests comparing between positive and negative infection samples (Fig. 6).

## Discussion

This study sheds new light on the gut bacterial microbiota of urban and rural populations in the Colombian Amazonian. In urban Leticia, we found lower bacterial diversity than in Km11 communities, reflecting reduced abundance of families related to non-processed foods (VANISH taxa), such as *Lachnospiraceae*, *Ruminococcaceae*, *Succinivibrio*, and *Spirochaetaceae*. The latter includes spirochetes of the genus *Treponema*, which are strongly associated with traditional rural populations of non-“Western” lifestyles [35]. Similar reductions in microbiota diversity have also been reported in other locations undergoing demographic and cultural transitions [53,54]. However, a noteworthy difference for Leticia is the increase of *Prevotella* instead of taxa associated with processed foods diets (BloSSUM taxa) in the urban setting.

Decreased microbial diversity in samples from the urban, mostly non-indigenous Leticia population compared to the rural, mostly indigenous Km11 population may be explained by lifestyle differences. Most of the Km11 donors practice activities such as small poultry farming for economic sustenance [55]. Outdoor activity [56] and livestock raising [57,58] have been shown to enrich microbiota diversity. Also, the diet in urban Leticia has changed significantly in recent decades compared to rural areas of the Amazonia, with reduced consumption of traditional non-industrialized foods like fish broth, wild animals, cassava-derived products like casabe and farinha, and fruit-based juices, and increased consumption of products such as packaged chicken, eggs, rice, canned foods, and powdered drink mixes of coffee, cocoa, or fruit [55].

Our comparative analyses show that the gut microbiota of people in and around Leticia is similar to that described within the Brazilian Amazonian urban and rural communities [4,6], with a high *Prevotellaceae* abundance and in general low presence of BloSSUM taxa. This pattern may be explained by similarities in the diet of these riverine communities along the Amazonian basin, which is still mostly composed of fish and polysaccharide-rich foods like cassava [4]. This differs from the lowland forest Tsimané community in Bolivia [34], the other Amazonian group evaluated, which has a diet rich in plant foraging and wild animals [59]. The taxonomic differences with Bogotá and Medellín, the other Colombian urban microbiotas, where BloSSUM taxa are more abundant, seem to reinforce the concept of a “tropical urban” category to describe microbiota of habitats of urban areas in tropical regions that are in different stages of microbiota “westernization” compared to non-tropical populations [4], although the different levels of urban transition, exemplified in the taxonomic differences between Leticia and Belém, indicate the necessity to further sample more Amazonian urban locations.

We also described the Leticia gut eukaryotic parasite community using a recently developed 18s rRNA metabarcoding protocol. We found a high abundance of intestinal parasites in both urban and rural samples, with *Endolimax* and *Entamoeba* presence having a positive effect on microbial richness and diversity. Infection with protists is already known to influence microbiota structure, being associated with higher microbial richness and abundance of VANISH taxa like *Prevotellaceae* and *Ruminococcaceae* [12,13]. We found that STH parasites are associated with higher bacterial microbial diversity and VANISH taxa abundance. A similar result, increased bacterial species diversity and *Prevotellaceae* abundance*,* was reported in a cohort of Colombian harboring mixed STH infections [60]. These results indicate potential parasite-microbiome interactions that could influence human health. This phenomenon has been extensively studied for both protists and helminths, which have been proposed to be beneficial by protecting against allergic and metabolic diseases [61–63].

Finally, predictive metabolic analysis indicates several significant results that may be relevant to health outcomes of the microbiota structure in the study area. Urban samples had relative depletion of fatty acid biosynthesis pathways. This finding may be explained by reduced abundance of *Lachnospiraceae*, which are key producers of butyrate and other short-chain fatty acids and decrease in diets high in saturated fatty acids [64]. Interestingly, depletion in short chain fatty acid producers was also observed in *Trichuris* infected individuals in a large study spanning Côte d’Ivoire, Laos, and Tanzania [65]. There was also an increase in the aerobic respiration pathways in urban samples, which may indicate increased saturated fatty acid consumption. These changes can increase the epithelial oxygenation in the colon, triggering a microbiome dysbiosis, characterized by an elevated abundance of facultatively aerobic bacteria compared to the healthy microbiota composition dominated by anaerobic bacteria [66]. Finally, although *Prevotellaceae* has been associated with a healthy microbiota, the high prevalence found in Leticia should be carefully interpreted, as this group has also been linked with inflammatory autoimmune diseases like rheumatoid arthritis [67].

Differences in the taxonomic composition and predicted physiology of the urban and rural Leticia microbiota documented in this study are likely associated with cultural and health transitions and intestinal protozoa and STH infections. These findings have relevance to public health, as such changes may underlie increases in chronic non-communicable diseases in the region, highlighting the need for further investigations into microbiota dynamics among urban and rural populations across the Colombian Amazon. This expanded analysis will be important for enhancing our understanding of the local health transitions and implementing proactive measures to improve public health outcomes and healthcare system preparedness.

## Supplementary Material

Supplement 1

## Figures and Tables

**Table 1:** Socio-economic and health parameters for Leticia and Km11 groups. PERMANOVA results for each parameter with p-value indicating statically significant influence on microbiota structure.

Socio-economic and health parameters	Permanova result	Parameter levels	Leticia (%)	Kilómetro 11 (%)
**Sex**	F = 0.56, R^2^ = 0.01 , p = 0.62	Female	60	59
		Male	40	41
**Age group**	F = 2.03, R^2^ = 0.04 , p = 0.08	1–15 yo	5	10
		16–30 yo	25	22
		31–85 yo	70	68
**Ethnic Identification**	F = 10.18, R^2^ = 0.10, p = 1.0E-3 ***	Indigenous	5	91
		Non-indigenous	95	9
**Scholarity level**	F = 1.97, R^2^ = 0.10 p = 0.02*	None	0	3
		Elementary school	20	27
		High school	25	60
		Technical school	40	6
		Undergraduate education	10	3
		Graduate education	5	1
**Ocupation**	F = 1.12, R^2^ = 0.05, p = 0.33	Unemployed	0	17
		Employee	65	14
		Independent	15	13
		Housewife	10	38
		Student	10	18
**Housing social strata** ^1^	F = 1.58, R^2^ = 0.05, p = 0.14	Rural	0	76
		Level one	20	21
		Level two	50	4
		Level three	30	0
**Number of house inhabitants**	F = 0.90, R^2^ = 0.02, p = 0.45	One	10	5
		Two to four	35	28
		Five or more	55	67
**Place where stay most of the day**	F = 0.86, R^2^ = 0.02, p = 0.52	Home	30	78
		Work/Chagra	70	21
		School	0	1
**Livestock contact** ^2^	F = 8.01, R^2^ = 0.08, p = 1.0E-3 ***	Yes	5	82
		No	95	18
**Companion animal contact**	F = 1.29 , R^2^ = 0.01, p = 0.21	Yes	55	22
		No	45	78
**Insecticide use**	F = 1.43 R^2^ = 0.08, p = 0.19	Yes	95	73
		No	5	27
**Mosquito net use**	F = 6.44, R^2^ = 0.06, p = 2.0E-3 **	Yes	25	94
		No	75	6
**Water storing at home**	F = 1.01, R^2^ = 0.01, p = 0.35	Yes	90	94
		No	10	6
**Healthcare coverage**	F = 3.37, R^2^ = 0 06, p = 0.01 **	No coverage	5	3
		Subsidized coverage	15	86
		Private coverage	80	12
**Hospitalization in the last year**	F = 0.39, R^2^ = 4.6E-3, p = 0.79	Yes	0	12
		No	100	88
**Chronic diseases history**	F = 1.49, R^2^ = 0.01, p = 0.18	Yes	35	22
		No	65	78
**Vector-borne diseases history**	F = 1.12, R^2^ = 0.01, p = 0.28	Yes	25	18
		No	75	82
**Chemiluminescence assay IgG SARS-CoV-2** ^3^	F = 1.06, R^2^ = 0.02, p = 0.32	Yes	55	58
		No	45	38
		Not measured	0	4
**Gen E RT-PCR SARS-CoV-2**	F = 0.81, R^2^ = 8.4E-3, p = 0.42	Yes	5	9
		No	95	91
**Medication use in the last month**	F = 2.39 , R^2^ = 0.02, p = 0.08	Yes	70	45
		No	30	55
**Smoking history**	F = 1.94, R^2^ = 0.02, p = 0.14	Yes	5	6
		No	95	94

1 Housing social strata based in the Colombian official strata divisions, rural and urban one to six levels.

2 Livestock contact includes raising and/or sacrificing domestic animals.

3 Three samples from Km11 were excluded because of the lack of biological material for testing.

## Relevance

Changes in the diversity and composition of gut microbiota in urban populations have been linked to the rise of non-communicable chronic diseases, such as autoimmune conditions, diabetes, and cancer. As developing countries undergo a demographic shift towards increased urbanization, accompanied by changes in diet, housing, and medication use, there is a concerning loss of microbial diversity. Therefore, it is essential to investigate microbiota changes in overlooked populations, such as indigenous communities in the Colombian Amazon basin. A better understanding of local and generalizable changes in gut microbial composition through urbanization may facilitate the development of targeted programs aimed at promoting lifestyle and diet changes, to prevent diseases that healthcare systems may be ill-equipped to effectively address.
