# Supplementary material for: Gut Microbiota and Parasite Dynamics in an Amazonian Community Undergoing Urbanization in Colombia
Source: medRxiv. 2025 Apr 17:2025.04.16.25325921. Preprint. [Version 1] doi: 10.1101/2025.04.16.25325921 (PMC12047915; doi:10.1101/2025.04.16.25325921)
Supplement: Supplement 1 [file NIHPP2025.04.16.25325921v1-supplement-1.pdf]

medRxiv preprint doi: https://doi.org/10.1101/2025.04.16.25325921; this version posted April 17, 2025. The copyright holder for this preprint (which was not certified by peer review) is the author/funder, who has granted medRxiv a license to display the preprint in perpetuity.
It is made available under a CC-BY 4.0 International license .

Table S1. Sample list for Amazonian and non-Amazonian datasets used for the comparative gut bacterial microbiota analysis.

Table S2. Overall abundance and logarithmic fold change value for OTUs with a differential abundance between Leticia and Km11 based on the Wald Significance Test.

Table S3. Metacyc database classification of the metabolic pathways with a differential abundance between Leticia and Km11 based on the Wald Significance Test.

Fig S1. Rarefaction curve for Leticia and Km11 samples.

Fig S2. Relative bacterial family abundance for Amazonian and non-Amazonian datasets with each bar summarizing the total of samples per dataset. Families with < 1% abundance were merged into one group.

medRxiv preprint doi: https://doi.org/10.1101/2025.04.16.25325921; this version posted April 17, 2025. The copyright holder for this preprint
(which was not certified by peer review) is the author/funder, who has granted medRxiv a license to display the preprint in perpetuity.
It is made available under a CC-BY 4.0 International license .

494 **Figure S1**

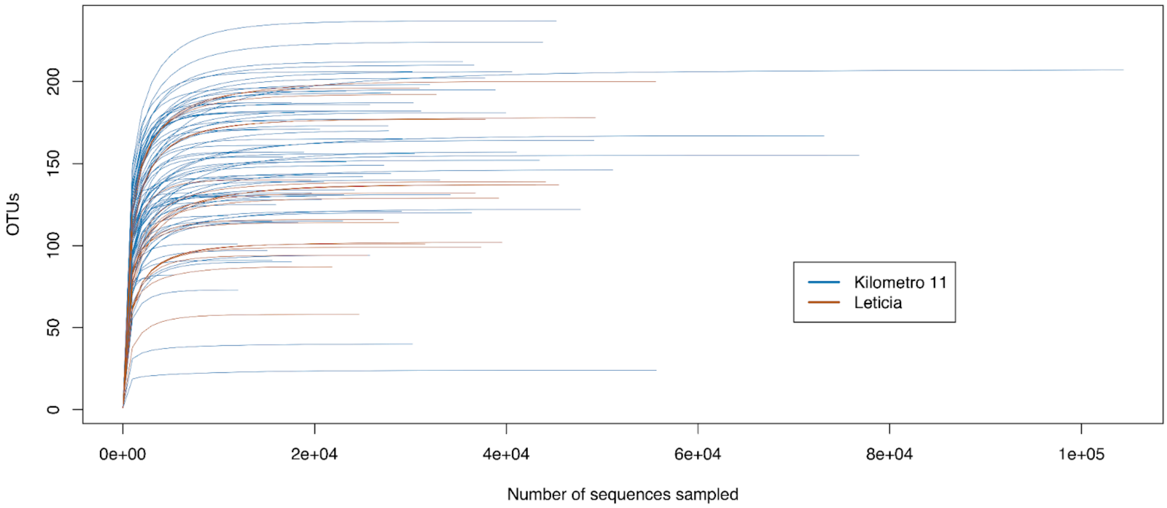

495

medRxiv preprint doi: https://doi.org/10.1101/2025.04.16.25325921; this version posted April 17, 2025. The copyright holder for this preprint
(which was not certified by peer review) is the author/funder, who has granted medRxiv a license to display the preprint in perpetuity.
It is made available under a CC-BY 4.0 International license .

496 **Figure S2**

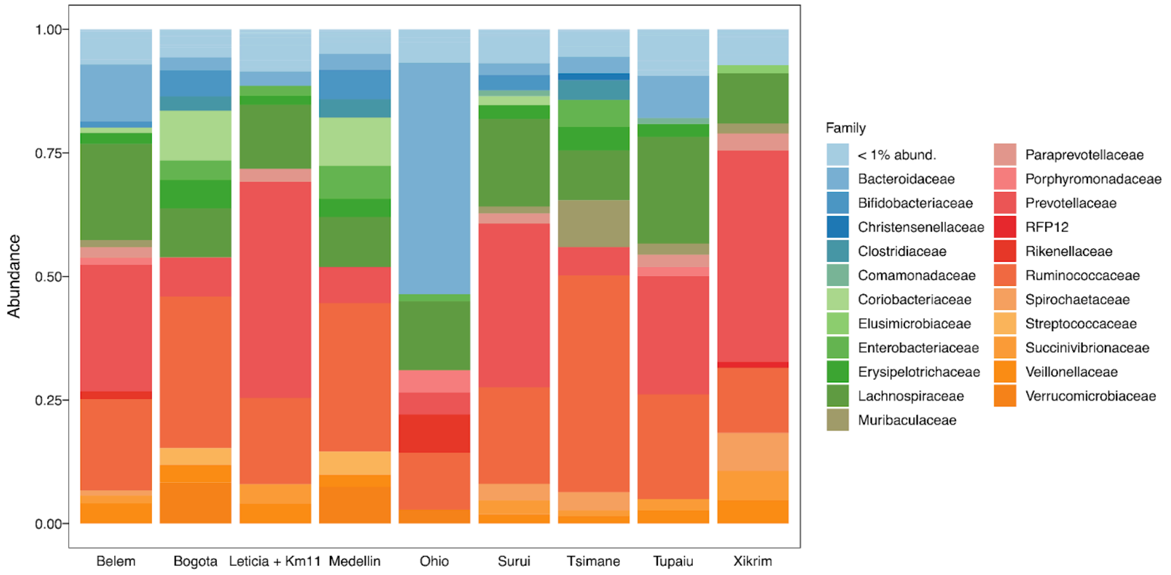

497
